# Sample Size Calculation: Inaccurate *A Priori* Assumptions for Nuisance Parameters Can Greatly Affect the Power of a Randomized Controlled Trial

**DOI:** 10.1371/journal.pone.0132578

**Published:** 2015-07-14

**Authors:** Elsa Tavernier, Bruno Giraudeau

**Affiliations:** 1 INSERM, U1153, Paris, France; 2 CHRU, Tours, France; 3 INSERM CIC 1415, Tours, France; 4 Université François-Rabelais de Tours, Tours, France; Utrecht University, NETHERLANDS

## Abstract

We aimed to examine the extent to which inaccurate assumptions for nuisance parameters used to calculate sample size can affect the power of a randomized controlled trial (RCT). In a simulation study, we separately considered an RCT with continuous, dichotomous or time-to-event outcomes, with associated nuisance parameters of standard deviation, success rate in the control group and survival rate in the control group at some time point, respectively. For each type of outcome, we calculated a required sample size *N* for a hypothesized treatment effect, an assumed nuisance parameter and a nominal power of 80%. We then assumed a nuisance parameter associated with a relative error at the design stage. For each type of outcome, we randomly drew 10,000 relative errors of the associated nuisance parameter (from empirical distributions derived from a previously published review). Then, retro-fitting the sample size formula, we derived, for the pre-calculated sample size *N*, the real power of the RCT, taking into account the relative error for the nuisance parameter. In total, 23%, 0% and 18% of RCTs with continuous, binary and time-to-event outcomes, respectively, were underpowered (i.e., the real power was < 60%, as compared with the 80% nominal power); 41%, 16% and 6%, respectively, were overpowered (i.e., with real power > 90%). Even with proper calculation of sample size, a substantial number of trials are underpowered or overpowered because of imprecise knowledge of nuisance parameters. Such findings raise questions about how sample size for RCTs should be determined.

## Introduction

When calculating the sample size for a randomized controlled trial (RCT) comparing two low-fat diets, Gardner *et al* [1] estimated 60 patients in each group, which corresponded to 80% power with two-sided type I error 5%, a hypothesized between-group difference in level of low-density lipoprotein cholesterol after 4 weeks of 0.26 mmol/L and an assumed standard deviation of 0.52 mmol/L. However, the observed standard deviation was 0.78 mmol/L. If we consider the real between-group difference equaling the hypothesized value, an estimated sample size of 60 patients in each group would ensure only 45% power for such a trial. To insure 80% power, the required sample size should have been 142 patients in each group.

This situation is not rare. Vickers [2], and more recently, Charles *et al* [3], found large discrepancies between values for *a priori*-assumed parameters used for sample size calculation and *a posteriori*-estimated ones from observed data. Vickers focused on the common underestimation of the nuisance parameter standard deviation with a continuous outcome and showed that the observed standard deviation was greater than the *a priori*-assumed standard deviation in 80% of RCTs. The same issues could arise when the outcome is binary, for which the success rate in the control group is *a priori*-specified.

Establishing a correct sample size is of utmost importance [4, 5] and the CONSORT Statement states that sample size calculations must be reported and justified in published articles [6]. If an RCT is too small, even with important differences among treatments, the trial results could be inconclusive (i.e., with no significantly statistical results). Moreover, even if the trial is conclusive, a too-small study would not be convincing enough to affect medical practice [7]. As well, patients included in too-small trials would not have the assurance that they were helping improve clinical practice, which raises ethical concerns [8]. Conversely, an RCT should be no larger than necessary. Indeed, an oversized trial would expose more patients than necessary to potential harm [9]. Such a trial would also lead to declaring a non-clinically relevant treatment effect as statistically significant [9]. Finally, it could consume more resources than necessary.

The aim of the present study was to assess how uncertainty in establishing nuisance parameters for continuous, dichotomous and time-to-event outcomes for RCTs affects the sample size calculations for trials and therefore real power.

## Materials and Methods

### Sample size calculation

In a superiority two-parallel-group RCT, the number of subjects is derived from four parameters: the hypothesized treatment effect on primary outcome measure, an assumption related to the control group, and the two *a priori*-specified statistical errors (considering that the type II statistical error is generally appraised with its complement, which corresponds to power). General equations for sample size calculations are in the S1 Appendix.

### Nuisance parameter

In real life, calculating a sample size is difficult because it depends on some parameter associated with the control group that has to be *a priori*-specified. Such a parameter is sometimes called a nuisance parameter [10]. We considered three types of data for a primary outcome: continuous, binary and time-to-event data. As shown in Table 1, each type of data is associated with a different way of hypothesizing a treatment effect but also implies assuming a nuisance parameter related to the control group. When the primary outcome is continuous, the treatment effect is often hypothesized as a mean difference. However, we also need to specify the standard deviation of the outcome (should be common to both the control and experimental groups). When the primary outcome is binary, we often hypothesize a success-rate difference, but we also have to assume the success rate expected in the control group. Finally, for a time-to-event outcome, the treatment effect is usually hypothesized as a hazard ratio or a survival-rate difference, but the expected survival rate in the control group at some time point must be *a priori*-specified for calculating the total number of patients to be included. These nuisance parameters are not the object of the study, but they need to be *a priori*-specified to plan the study. *A priori*-specified values are usually derived from previously published data. The quality of the assumption for a nuisance parameter made at this step relies heavily on the precision of the parameters derived from previous studies as well as similarities of these previous studies to the one being planned in terms of population. An incorrect assumption for a nuisance parameter could affect the power of the planned study (thus leading to an underpowered or overpowered study), and the aim of the present work was to assess the extent of such an impact.

**Table 1:** Parameter of interest and nuisance parameter for the different types of data.

**Type of data**	**Hypothesized treatment effect**	**Nuisance parameter**
Continuous	Mean difference	Standard deviation in the control group
Binary	Success rate difference	Success rate in the control group
Time-to-event	Hazard ratio	Survival rate in the control group at some time point

### Simulation study

To assess the impact of errors made in *a priori*-specifying a nuisance parameter on power in RCTs, we performed a simulation study. For each of the three types of outcomes, we considered a two-parallel-group RCT for which we hypothesized a treatment effect. We also assumed a nuisance parameter in the control group. With the chosen values, we calculated the appropriate sample size N considering 80% power, with two-sided type I error 5%. Then we considered that the true nuisance parameter differed from the *a priori*-assumed nuisance parameter. For each simulation, we used a probability density function (cf infra) to determine the error made in the nuisance parameter by comparing the assumed and the deduced true nuisance parameter. Considering the previously calculated sample size of N, we then retro-fitted the sample size formula to derive the real power of the trial with the true nuisance parameter to detect the treatment effect that had been hypothesized. Such a procedure was re-run 10,000 times to allow for estimating the proportion of underpowered or overpowered RCTs. The same simulation study was repeated considering a nominal power of 90%. Full details of the algorithms used are in the S2 Appendix.

### Relative errors on nuisance parameters

**Fig 1:**
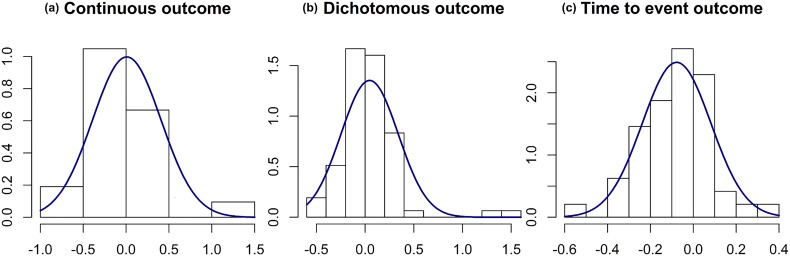
Distribution curves fitted on the relative errors observed for nuisance parameters. A gamma distribution was fitted for continuous outcomes. Angular transformations were applied before calculating relative errors for dichotomous and time-to-event outcomes, then normal distribution curves were fitted. Dataset of 147 published trials. (a) Relative error between the observed standard deviation compared to the postulated standard deviation for continuous data on for studies. (b) Relative error between the observed rate in the control group compared to the postulated rate in the control group for dichotomous data for 78 studies. (c) Relative error between the observed rate in the control group compared to the postulated rate in the control group for time to event data for 48 studies

To simulate the errors made in the *a priori*-specified nuisance parameter, we used data from a survey of 215 RTCs published in 2004 and 2006 in 6 general journals with high impact factor. In this survey, Charles *et al* [3] compared the assumptions in nuisance parameters made for the sample size calculation and the results. Data extracted included *a priori*-specified nuisance parameters and corresponding observed values reported in results sections. The *a priori*-specified assumptions and observed values could be compared for 147 articles reporting enough information (namely, in the sample size calculation section). For the three types of data, we estimated the relative difference between the observed parameter and its *a priori*-specified value (i.e., the difference between the observed minus the *a priori*-specified value divided by the *a priori*-specified value). We performed this calculation for 21 standard deviations, 78 success rates and 48 survival rates. Moreover, for success rates and survival rates, we applied an angular transformation to stretch out the values near 0 or 1 before calculating relative differences. Histograms of these relative differences are in Fig 1. A distribution was fitted for each of the three types of data. For continuous outcomes, the mean relative difference between the observed standard deviation of the outcome and its *a priori*-specified value was 0, with standard deviation 0.4. For binary outcomes, the mean relative difference between the observed success rate in the control group and its *a priori*-specified value was 0.05, with standard deviation 0.3. Finally, for time-to-event outcomes, the mean relative difference between the observed survival rate in the control group and its *a priori*-specified value was -0.1, with standard deviation 0.2.

## Results

**Fig 2:**
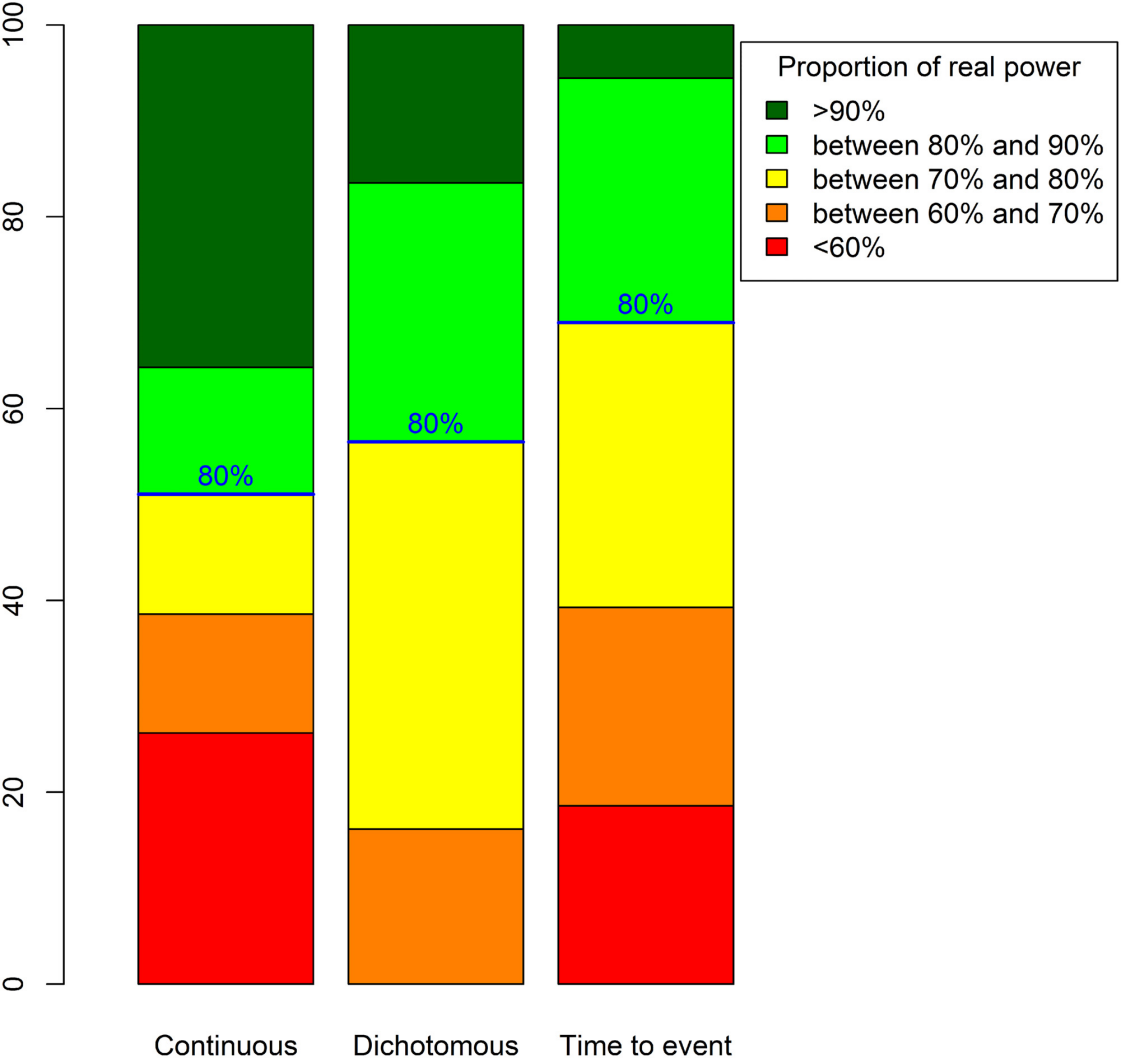
Real power distributions for 80% intended power and considering a normal distribution for the relative error for the true nuisance parameter.

Fig 2 displays real power proportions for the three types of data. For trials with continuous outcomes, 23% had a real power < 60%. Therefore, for one quarter of such RCTs, the planned sample size should be increased by at least 60% (cf S3 Appendix). However, 41% of such trials had a real power > 90%, so in these trials, the planned sample size was 25% greater than would be necessary. For trials with dichotomous outcomes, no RCT had a power < 60%, but 16% had a real power > 90% and would thus include at least 25% more patients than necessary. Finally, for trials with time-to-event outcomes, 18% had a real power < 60%, so the sample size should be increased by at least 60% to reach the nominal power of 80%. Only a few of these RCTs (6%) had a real power > 90%, so the planned number of recruited patients was overestimated by about 25%.

The discrepancies between real power and nominal 80% power were greater for continuous than dichotomous or time-to-event outcomes, which agrees with the distribution of relative errors having more widespread distribution for continuous than other outcomes. We presume that this finding is due to the greater complexity in specifying a standard deviation than a success rate or survival rate: the latter parameters are indeed more concrete and understandable than the former parameter. Finally, we found a certain proportion of underpowered trials with time-to-event outcomes, which is explained by the non-centered distribution of the relative error associated with the *a priori*-specified rate of events in the control group.

When the nominal power is 90%, 12.7% of trials with continuous outcomes and 5.3% of those with time-to-event outcomes had a real power of < 60%. The proportion of overpowered trials (> 90% power) was 54% for those with continuous outcomes, 42.8% for those with dichotomous outcomes and 31.6% for those with time-to-event outcomes.

## Discussion

The present simulation study illustrates how realistic errors in assumptions for nuisance parameters translates into decreased or increased real power of an RCT. Within the parameters of the simulation, a large proportion of RCTs (i.e., 23% and 18% of RCTs with continuous and time-to-event outcomes, respectively) had a real power < 60%, that is, far less than the nominal 80% value. In contrast, overpowered RCTs were also common: 41%, 16% and 6% of RCTs with continuous, binary and time-to-event outcomes, respectively, had real power > 90%. When considering a higher nominal power (90%), the proportion of trials with power < 60% decreased to 12.7% for those with continuous outcomes and 5.3% for those with time-to-event outcomes. Our study has one limitation in that the distribution of the relative errors for the nuisance parameters were derived from a limited set of articles. Nevertheless, these distributions are realistic because they are derived from real RCTs.

Several explanations may explain the discrepancies between *a priori*-specified assumptions and observed values for nuisance parameters. The discrepancies may be due to lack of precision in estimating these nuisance parameters in previously published studies, caused by the nuisance parameters being sample estimates but assumed to be known in the sample size calculation. Moreover, trialists may also base their assumptions on monocenter pilot studies. In doing so, one may face another limitation: heterogeneity is less likely between patients from a common center than patients from different centers. Especially for continuous outcomes, standard deviations derived from monocenter pilot studies are expected to underestimate standard deviations derived from multicentric studies [2].

A targeted power of 90% instead of 80% allows for decreasing the proportion of trials with real power < 60%. As well, a study with a targeted 90% power will much more likely ensure at least the acceptable level of 80% than a study with a targeted 80% power. This consideration is related to the errors made on the nuisance parameter. Indeed, trials with 90% power are more likely to recruit for at least 80% power than those planned for 80% power [11]. Therefore the choice of 90% power is preferable when performing a sample size calculation. Moreover, some methods exist to deal with uncertainty associated with nuisance parameters. Thus, when planning the trial, the sensitivity of the sample size calculation to the imprecision of the population variance estimate [12, 13], or to the population success rate estimate for the control groups [14] should be investigated. Although such methods are rarely used (Clark et al. observed they were used in only 3% of a series of 446 protocols [15]), we could surely take advantage of using them more frequently. Otherwise, during the trial, sample re-estimations [16, 17], also referred to as internal pilot studies [18], are a kind of adaptive design. The idea is to consider part of the main trial as a pilot phase. The design is used for recomputing the nuisance parameter and recalculating the required sample size during the trial. The final analyses then incorporate all data, ignoring that part of the data came from a pilot phase. In this scenario, the first few patients entered in the trial should be more representative of the population of the trial than patients from a previous pilot study. However, such an approach has some limitations. First, it supposes an outcome rapidly assessed after randomization, thus allowing a sample size re-estimation before recruitment has ended. Second, there is a high risk of great imprecision with re-estimating nuisance parameters, which could be highly damaging. Finally, the approach supposes that the trialists have enough resources to increase the sample size, which is not always the case, namely for publicly funded RCTs.

Our results also illustrate that the emphasis on sample size calculation is inconsistent with the major difficulties that inevitably come with it, which explains why the usual guidance has been criticized. Bacchetti holds such calculations as responsible for the “threshold myth” [19]. In other words, current conventions assume a meaningful demarcation at which sample sizes are considered fair and adequate and that any smaller sample size would imply a wasted trial: this illusion causes substantial harm to the research process and he encourages alternative approaches based on cost and feasibility [20, 21]. As well, Norman *et al* [22] supports the use of “off-the-peg” sample sizes when sufficient information is not available for a “made-to-measure” calculation. In general, authors agree with Bacchetti on the need to determine a sample size by more than literal statistical calculations.

Besides sample size issues, a unique trial often cannot provide definite evidence for the existence or absence of a treatment effect. Misinterpretations are indeed likely with the use of p values to dichotomise significant or non-significant results, which indicates the need for both small and large studies to focus on confidence intervals rather than p values [23]. Moreover systematic reviews and meta-analyses combining evidence from several RCTs are considered a higher level of evidence. Altough results from a single, small, underpowered trial may be unreliable, some authors nevertheless consider such trials legitimate [5, 24]. These trials are indeed expected to contribute to a body of knowledge, and a forthcoming meta-analysis can give a definite answer [25]. Indeed, with little data available, starting small seems meaningful and relevant [21]. Moreover, results from meta-analyses are more informative in that they allow for appraising the variability in between-RCT treatment effects and are a good opportunity to explore such variability.

These issues confirm and reinforce the fundamental idea that results from one trial should not be interpreted alone. In the end, the present work illustrates once again [3] the discrepancy between the important emphasis put on the sample size calculations and the reality of the great imprecision when implementing them.

## Supporting Information

S1 Appendix Sample size calculations. (PDF)

S2 Appendix Simulation study. (PDF)

S3 Appendix How an over- or underpowered trial translates to under- or overrecruitment. (PDF)

S1 Text Powers for continuous outcomes over 10,000 simulations. (TXT)

S2 Text Powers for binary outcomes over 10,000 simulations. (TXT)

S3 Text Powers for time-to-event outcomes over 10,000 simulations. (TXT)
