# Supplementary material for: Sample Size Calculation: Inaccurate A Priori Assumptions for Nuisance Parameters Can Greatly Affect the Power of a Randomized Controlled Trial
Source: PLoS One. 2015 Jul 14;10(7):e0132578. doi: 10.1371/journal.pone.0132578 (PMC4501786; doi:10.1371/journal.pone.0132578)
Supplement: S1 Appendix — (PDF) [file pone.0132578.s001.pdf]

S1 Appendix

Sample size calculations. Considering a continuous outcome, the *a priori* hypothesis is a mean difference. Moreover, trialists need some assumption regarding the standard deviation of the outcome (often derived from previous studies) and set values for both the type I error (i.e., α) and the nominal power of the trial (i.e., 1-β). If we let δ be the mean difference and σ the standard deviation of the outcome, the number of patients to be recruited in each group is [1]:

$$n = \frac{2(Z_{\frac{\alpha}{2}} + Z_{\beta})^2}{\Delta^2}$$

with the effect size

$$\Delta = \frac{\delta}{\sigma}$$

and Z_q is the q-percentile of a standard normal distribution.

If the outcome is dichotomous (e.g., a success rate), the clinical hypothesis is a rate difference. Moreover, we have to specify the rate associated with the control group. If we define and p_C and p_T as the success rate in the control and interventional groups, respectively, the number of patients to be recruited in each group could be calculated as [2]:

$$n = \frac{2(Z_{\frac{\alpha}{2}} + Z_{\beta})^2}{\Delta^2}$$

with

$$\Delta = \frac{p_T - p_C}{\sqrt{\frac{p_T(1-p_T)+p_C(1-p_C)}{2}}}$$

Finally for time-to-event data, sample size is usually derived considering survival rates at some time point. Thus, assuming that the survival proportions is π_C in the control group and π_T in the treatment group, at some chosen time, the *a priori* hypothesis is the hazard ratio defined as $HR = \frac{\log \pi_T}{\log \pi_C}$. The number of patients to be recruited in each group is then [3]:

$$n = \frac{\left(\frac{HR+1}{HR-1}\right)^2 (Z_{\frac{\alpha}{2}} + Z_{\beta})^2}{2 - \pi_T - \pi_C}.$$

References

[1] Julious SA (2004) Sample sizes for clinical trials with normal data. Statistics in Medicine 23: 1921–1986.

[2] Julious SA, Campbell MJ (2012) Tutorial in biostatistics: sample sizes for parallel group clinical trials with binary data. Statistics in Medicine 31: 2904–2936.

[3] Machin D, Campbell MJ, Tan SB, Tan SH (2008) Sample Size Tables for Clinical Studies. Chichester, West Sussex, UK ; Hoboken, NJ: Wiley-Blackwell, 3rd edition edition.
