# Supplementary material for: Sample Size Calculation: Inaccurate A Priori Assumptions for Nuisance Parameters Can Greatly Affect the Power of a Randomized Controlled Trial
Source: PLoS One. 2015 Jul 14;10(7):e0132578. doi: 10.1371/journal.pone.0132578 (PMC4501786; doi:10.1371/journal.pone.0132578)
Supplement: S2 Appendix — (PDF) [file pone.0132578.s002.pdf]

S2 Appendix

Simulation study.

Continuous outcomes We hypothesized a difference in means of $\delta = 2$ between the two groups. We also assumed a standard deviation $\sigma^{assum} = 10$ in the control group, which led to a hypothesized effect size of $2/10 = 0.2$. With such values and considering 80% power with two-sided type I error 5%, the required sample size was 393 patients in each group. Then we considered that the true standard deviation σ differed from the assumed standard deviation σ^{assum}. Then considering the relative error distribution in Fig. 1.a (i.e., with mean $\mu_\epsilon = 0$ and standard deviation $\sigma_\epsilon = 0.4$), we performed the following steps:

1. We randomly generated a value of the relative error $\epsilon \sim \Gamma(k, \theta) - 1$ with $k\theta = \mu_\epsilon + 1$ and $k\theta^2 = \sigma_\epsilon^2$
2. The true standard deviation was deduced as $\sigma = \sigma^{assum} \cdot (1 + \epsilon)$
3. The true difference in means remained identical to the hypothesized value $\delta = 2$
4. Considering a sample size of 786 patients, we derived the power of such a trial to detect a difference of δ with standard deviation σ

Steps 1 to 4 were repeated 10,000 times.

Binary outcomes We hypothesized a difference in success rates of $\delta = 10\%$ between the two groups and assumed a rate $p_C^{assum} = 20\%$ in the control group. With such values, and considering 80% power with two-sided type I error 5%, the required sample size was 290 patients in each group. Then, considering the relative error in Fig. 1.b (i.e., with mean $\mu_\epsilon = 0.05$ and standard deviation $\sigma_\epsilon = 0.3$),

1. We randomly generated a value of the relative error $\epsilon \sim \mathcal{N}(\mu_\epsilon, \sigma_\epsilon)$
2. The true rate in the control group was deduced from $arcsin(\sqrt{p_C}) = (1+\epsilon)arcsin(\sqrt{p_C^{assum}})$ because we applied an angular transformation before calculating relative differences.
3. The true difference in rates remained identical to the hypothesized value $\delta = 10\%$
4. Considering a sample size of 580 patients, we derived the power of such a trial to detect a difference of δ with a success rate for the control group of p_C

Steps 1 to 4 were repeated 10,000 times.

Time-to-event outcomes We hypothesized a hazard ratio $HR = \frac{\log 0.8}{\log 0.7} = 0.63$ with the assumption of the probability of events $p_C^{assum} = 30\%$ in the control group. With such values, and considering 80% power with two-sided type I error of 5%, the required sample size was 296 patients in each group. Then, considering the relative error in Fig. 1.c (i.e., with mean $\mu_\epsilon = -0.1$ and standard deviation $\sigma_\epsilon = 0.2$),

1. We randomly generated a value of the relative error $\epsilon \sim \mathcal{N}(\mu_\epsilon, \sigma_\epsilon)$
2. The true probability of events in the control group was deduced from $arcsin(\sqrt{p_C}) = (1+\epsilon)arcsin(\sqrt{p_C^{assum}})$ because we applied an angular transformation before calculating relative differences.

3. The true hazard ratio remained identical to the hypothesized value HR
4. Considering a sample size of 592 patients, we derived the power of such a trial to detect a hazard ratio of HR with the probablilty of event in the control group of p_C

Steps 1 to 4 were repeated 10,000 times.
