# Supplementary material for: Sample Size Calculation: Inaccurate A Priori Assumptions for Nuisance Parameters Can Greatly Affect the Power of a Randomized Controlled Trial
Source: PLoS One. 2015 Jul 14;10(7):e0132578. doi: 10.1371/journal.pone.0132578 (PMC4501786; doi:10.1371/journal.pone.0132578)
Supplement: S3 Appendix — (PDF) [file pone.0132578.s003.pdf]

S3 Appendix

How an over- or underpowered trial translates to under- or overrecruitment.

Continuous outcomes Considering a difference in means of $\delta = 2$ between the two groups, standard deviation $\sigma = 10$ in the control group, type I error 5% (i.e., α) and 80% power (i.e., 1-β), we derived the required sample size of $n = 393$ patients from the following equation:

$$n = \frac{2(Z_{\frac{\alpha}{2}} + Z_{\beta})^2}{(\delta/\sigma)^2}.$$

From the same equation, we can deduce the standard deviation $\delta = 2$, $\alpha = 0.05$ and $\beta = 0.4$ (ie 60% power), which is $\sigma_{60\%power} = 12.67$. Because the power decreases at higher standard deviations, if 26% of the trials have a real power $< 60\%$, then 26% of the trials have a real standard deviation greater than $\sigma_{60\%power}$. Considering a difference in means of $\delta = 2$ between the two groups, standard deviation $\sigma = \sigma_{60\%power}$ in the control group, type I error 5% and 80% power, we derived a required sample size of 630 patients in each group, representing a 60% increase in sample size from the 393 initially calculated. Similarly, if 36% of the trials have a real power $> 90\%$, then 36% of the trials have a real standard deviation less than $\sigma_{90\%power} = 8.65$, which corresponds to 294 patients in each group, so the planned sample size is 25% greater than would be necessary.

Binary outcomes As described for continuous outcomes, with a difference in rates of 10% between the two groups and a type I error of 5%, if 16% of the trials have a power $> 90\%$, then 16% of the trials have a real success rate in the control group lower than $p_{C,90\%power} = 0.119$. Considering 80% power between the two groups, this corresponds to 217 patients in each group, so the planned sample size is 25% greater than would be necessary.

Time-to-event outcomes As described for continuous outcomes, with a hazard ratio of $HR = \frac{\log 0.8}{\log 0.7} = 0.63$ between the two groups and type I error 5%, if 18% of the trials have a power $< 60\%$ then 18% of the trials have a real probability of events in the control group lower than $p_{C,60\%power} = 0.189$. Considering 80% power between the two groups, this corresponds to 475 patients in each group, representing a 60% increase in sample size. Likewise, if 6% of the trials have power $> 90\%$, then 6% of the trials have a real probability of events in the control group greater than $p_{C,90\%power} = 0.398$. Considering 80% power between the two groups, this corresponds to 221 patients in each group, so the planned sample size is 25% greater than would be necessary.
